# Neuromuscular Ultrasound in the Pediatric Population

**DOI:** 10.3390/diagnostics10121012

**Published:** 2020-11-26

**Authors:** Trent A. VanHorn, Michael S. Cartwright

**Affiliations:** Department of Neurology, Wake Forest School of Medicine, Winston-Salem, NC 27157, USA; tvanhorn@wakehealth.edu

**Keywords:** ultrasound, pediatric, nerve ultrasound, muscle ultrasound, diagnostic

## Abstract

The diagnosis and evaluation of neuromuscular disorders traditionally involves electrodiagnostic (EDx) testing, including nerve conduction studies (NCSs) and electromyography (EMG). These tools can cause pain and discomfort, an important consideration when performed on children. Neuromuscular ultrasound is noninvasive, cost-effective, and increasingly utilized for the detection of neuromuscular pathology. Studies investigating the performance and clinical implementation of ultrasound have primarily been performed in adult populations. Ultrasound in children has the potential to guide EDx testing and ultimately improve diagnostic efficiency and accuracy. This review aims to describe key features of neuromuscular ultrasound in the pediatric population based on the available studies, including our own institutional experience.

## 1. Introduction

The approach to the evaluation and diagnosis of neuromuscular pathologies has evolved over the past several decades. Electrodiagnostics (EDx), including nerve conduction studies (NCSs) and electromyography (EMG), inform clinical decision-making by providing valuable neurophysiologic information. In addition to these well-established modalities, neuromuscular ultrasound has emerged as an important complementary test in both adult and pediatric populations [1,2,3,4,5,6]. Importantly, most of the research has been conducted in adults. This review aims to describe key features of neuromuscular ultrasound in the pediatric population based on the available studies, including our own institutional experience. It is important to note that other imaging modalities, such as magnetic resonance imaging (MRI), have also been used in the assessment of pediatric neuromuscular conditions. While MRI has some advantages, such as variable sequences to differentiate tissues, ultrasound also has advantages, including point-of-care delivery, dynamic imaging, and higher resolution [7]. All imaging techniques continue to improve rapidly, and the future may include multiple combined imaging techniques to obtain the most accurate neuromuscular diagnosis.

## 2. Testing Considerations

Performing electrodiagnostic testing differentially impacts pediatric patients when compared with adults. Specifically, the inability to tolerate pain and discomfort may limit comprehensive testing in this group. Significant contributors to increased pain include examination of proximal muscle groups, >1 muscles sampled, and the performance of EMG and NCS compared to NCS alone [8]. Overall, self-reported pain scores are rated as moderately intense for both NCS and EMG. In a study of 498 children and adolescents, the age of the patient was the main factor determining whether a useful result was obtained from EDx testing [8]. Testing performed on patients less than three years of age is more likely to result in inadequate or incomplete findings. In addition, the inability to follow commands in young patients is a barrier in some contexts. For example, when performing testing on infants with hypotonia, recording muscle action potentials during volitional movement can be challenging. Neuromuscular ultrasound is not similarly impacted by pain and discomfort, making it an attractive tool for young patients who cannot meaningfully participate in EDx testing or who are unable to tolerate it. 

While procedural difficulties associated with EDx testing in younger patients are important considerations, the overall value and accuracy of electrodiagnostic testing remains high and significantly informs decision-making. An under-recognized value of neuromuscular ultrasound is the potential to increase the likelihood of successful EDx based on sonographic findings and guidance. Additional literature demonstrating consistency related to this benefit is needed. 

In addition to ultrasound, computed tomography (CT) and MRI can also image nerve and muscle. However, there are limitations to these techniques. CT and, in particular, MRI require children to remain still during imaging, which may require anesthesia. This introduces a risk that is not present when imaging with ultrasound. In addition, imaging with these modalities does not generate the high resolution that one can obtain with ultrasound. While MRI approaches the resolution of ultrasound, high-frequency ultrasound still results in higher resolution and more accurate diagnoses than MRI [9].

## 3. Nerve Ultrasound in Children 

For accurate interpretation of nerve ultrasound in the pediatric population, clinicians must consider the impact of age on the developing peripheral nervous system. While normal nerve cross-sectional areas (CSAs) have been defined at distinct locations in the adult population, the available data for children and adolescents continues to mature. A correlation certainly exists between nerve CSAs and age [10,11]. Nerve size continues to increase until adult size values are reached at around 15–17 years of age [10,11,12]. Defining age-specific reference values is critical for the interpretation of quantitative sonographic findings and for subsequent comparison across groups. A normative CSA range may be applied across multiple ages with adequate accuracy i.e., ages 2–4. However, age-specific values are likely necessary during teenage years to account for the increased rate of growth seen during this period [12,13]. Age ultimately becomes a poor predictor of CSAs following teenage years. Previous studies have investigated the relationship between the CSA and weight, body mass index, and height in healthy individuals [10,14,15,16,17]. Beyond the period of early nerve growth, nerve size appears to correlate most closely with weight, although the literature does not offer definitive evidence for the variable most closely associated with CSAs measured by ultrasound [18].

In young patients with pathology affecting the peripheral nerves, more sonographic data is needed before clear recommendations can be offered concerning the diagnostic utility of this noninvasive imaging modality. Sonographic evidence of peripheral nerve enlargement has been described in entrapment neuropathies, inherited and acquired polyneuropathies, trauma, and tumors [10,11,12]. As most of these conditions are more frequently seen in adults, a definitive approach to evaluating potential nerve enlargement in pediatric patients has been challenging to describe. Zaidman et al. investigated sonographic nerve enlargement in acquired and inherited polyneuropathies, and found that age did not impact the pattern or degree of nerve enlargement in patients with Charcot-Marie-Tooth 1A (CMT-1) [18]. In order to make direct comparisons between individuals of varying ages, differences in expected CSAs were accounted for by height, based on previous work by the same group demonstrating nerve size correlated with height [12]. Other studies have also described nerve enlargement in children with inherited demyelinating polyneuropathies and inflammatory neuropathies [19]. In cases of acquired disorders, such as chronic inflammatory demyelinating polyneuropathy (CIDP), Guillain-Barré syndrome, and multifocal motor neuropathy, where nerve enlargement is regionally present, age differences have not revealed varying patterns of nerve involvement [20].

Importantly, sonographic measurements of pathologic peripheral nerves in children may fall within the normal range for CSAs in adults. This potential pitfall underscores the importance of interpreting CSAs within the context of age-defined reference values [13]. Additionally, intra- and inter-nerve comparisons can be very helpful in children when trying to determine if a single site or nerve is enlarged, as can other variables, such as fascicle size, echogenicity, and vascularity. When evaluated by ultrasound, pathologic changes to the peripheral nerves appear similarly in pediatric patients when compared to adults. The established diagnostic utility of this tool in adult patients is unlikely diminished by the age of the patient, and should not be seen as a barrier to its use. 

There are several specific disease states in which nerve ultrasound is beneficial. Focal mononeuropathies are the conditions most commonly studied with neuromuscular ultrasound, and ultrasound has proven highly effective for diagnosis in these conditions [21]. Mononeuropathies are less common in children than adults, and therefore, these conditions in children often require more investigation than in adults. Ultrasound is helpful for identifying carpal tunnel syndrome in mucopolysaccharidoses, lipofibromatous hamartomas, brachial plexopathies, and other traumatic mononeuropathies [22,23,24,25]. More common in children are polyneuropathies, which may be hereditary or acquired. Nerve ultrasound in children has been shown to assist in the differentiation of CMT from inflammatory polyneuropathies, with CMT typically showing uniform enlargement of all nerves and acquired inflammatory processes showing patchy nerve enlargement [26]. In addition, nerve ultrasound has shown that some hereditary polyneuropathies have features similar to acquired processes, which may have treatment implications [27]. Further research regarding nerve ultrasound in children should include assessment of fascicle size and number, echotexture, and elastography. 

## 4. Muscle Ultrasound in Children

Muscle ultrasound is a noninvasive, inexpensive, non-painful, and reproducible tool that is increasingly utilized as a supplement to serum creatine kinase, EMG, muscle histology, immunohistochemistry, and genetic analyses in the evaluation of children and adults with suspected neuromuscular pathology. Similar to nerve ultrasound, the potential influence of age and maturation on muscle ultrasound has been investigated. Importantly, neither muscle thickness nor echo intensity are dependent on age based on quantitative ultrasound assessment in healthy children. This determination underscores the value of identifying increased echogenicity on muscle ultrasound, as it is most likely due to pathological changes in the muscle and likely a more accurate parameter than thickness alone [28]. Importantly, the predictive value of muscle ultrasound is diminished in children less than three years of age, before significant structural changes, fat proliferation, or fibrosis have developed [28,29]. Overall, high accuracy has been reported in detecting the presence of muscle pathology by sonographic changes. Sensitivity and specificity related to the tool continue to improve with the maturation of quantitative and qualitative sonographic measurements and the ability to compare data across ultrasound devices. In general, the literature supports the use of muscle ultrasound in children as an initial screening when neuromuscular pathology is suspected [28,29]. 

Although more challenging, the use of muscle ultrasound to differentiate myopathic from neurogenic conditions continues to expand as increasingly more prospective data describing sonographic features in specific neuromuscular pathologies becomes available [19]. This determination can ultimately be made with high sensitivity and specificity based on the pattern of pathologic muscle involvement identified by ultrasound [19,20]. The positive predictive value of muscle ultrasound in conditions such as muscular dystrophy and inflammatory myopathies exceeds 90%, which is likely a reflection of the high degree of histopathological changes seen in these conditions. Even in metabolic myopathies, such as Pompe disease, muscle ultrasound can serve as an appropriate initial screening test [4,30,31]. Obtaining ultrasound imaging prior to invasive and painful testing can inform clinicians as to which muscles may offer highest yield in terms of EMG sampling and subsequent biopsy location [20]. In the era of next generation sequencing, the added data provided by muscle ultrasound becomes increasingly informative, particularly when tasked with diagnosing congenital muscle diseases, and it can guide genetic testing choices and serve as a biomarker of disease progression [30].

Many specific myopathic processes have been studied with neuromuscular ultrasound. These include congenital muscular dystrophies, muscular dystrophies, congenital myopathies, metabolic myopathies, inflammatory myopathies, and primary nerve diseases, such as spinal muscular atrophy (SMA) [32,33,34,35]. When using ultrasound to assess potential myopathies, important considerations include the pattern of muscle involvement and the presence of a Doppler signal, as inflammatory myopathies are often associated with an increased Doppler signal in the muscle and/or associated fascia. In general, myopathies show homogeneously increased muscle echogenicity (ground glass appearance), whereas neuropathies show patchy increased muscle echogenicity. Muscle ultrasound has the potential to serve as a biomarker of disease progression in both primary muscle and nerve disease. For example, muscle ultrasound, measuring thickness and quantitative echogenicity, can be used to follow children with SMA, which is of particular interest now that this condition can be treated with gene replacement and antisense oligonucleotides [36]. Of note, ultrasound can even be used to guide lumbar punctures for intrathecal treatment in SMA [37]. Future research into muscle ultrasound should include newer parameters, such as elastography and echotexture, increased resolution, Doppler changes, three-dimensional imaging, and defining patterns of muscle involvement. 

## 5. Neuromuscular Ultrasound in an Electrodiagnostic Lab

Recommendations to incorporate, supplement, or replace traditionally utilized diagnostic modalities with neuromuscular ultrasound have been made for conditions such as carpal tunnel syndrome and other focal neuropathies [38,39,40]. In addition, the cost-effectiveness associated with ultrasound has been well documented, especially when compared to other imaging modalities, such as MRI [41]. Protocols involving neuromuscular ultrasound as a diagnostic tool in the pediatric population have not been extensively described in the literature. Practically, performing muscle ultrasound at several locations prior to needle EMG is quick and painless [42,43]. At our institution, incorporation of neuromuscular ultrasound as the first diagnostic test within the electrodiagnostic lab resulted in significantly fewer NCS and EMG muscle samples required to reach a diagnosis in pediatric patients [5]. Ultrasound findings helped guide subsequent EMG and biopsy sampling. Remarkably, in our sample, imaging led to positive EDx testing of muscle groups that were not initially suspected as sites of pathology based on physical exam or locations commonly associated with a suspected pathology. Efficient incorporation of neuromuscular ultrasound ultimately relies on clinicians trained in performing and interpreting sonographic findings within an electrodiagnostic lab, a potential limitation to its widespread use. In addition, low awareness surrounding the evolving role and utility of neuromuscular ultrasound on the part of clinicians evaluating peripheral neuropathies is a potential barrier to increased implementation. However, neuromuscular ultrasound can be quickly learned, and is an ideal technique for neurologists and physiatrists accustomed to evaluating neuromuscular pathology [44]. In our laboratory, sedation is not used to perform neuromuscular ultrasound. Most children tolerate the procedure very well, and although children under age two years may require some restraint from their parent, sedation and anesthesia are not needed. 

## 6. Illustrative Case

**Figure 1:**
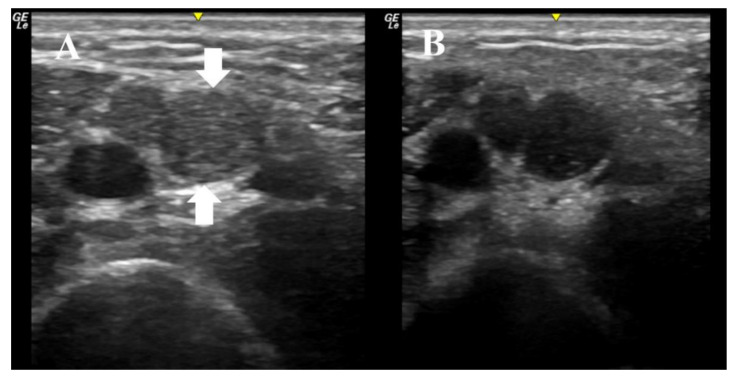
These ultrasound images were obtained with a 22 MHz linear array transducer, and they depict the median nerve in the right arm of an 11-year-old boy with subacute distal weakness and sensory loss; (**A**) demonstrates imaging with the transducer at 90 degrees to the nerve. The nerve has two large fascicles, one between the arrows and the other directly to the left of the largest fascicle. The area of the nerve at this site is 20 mm^2^. (**B**) demonstrates the same image, but the transducer is at an angle just slightly off 90 degrees, which demonstrates the anisotropy of the nerve at this site, as the fascicles become hypoechoic with just a minor alteration of the angle of insonation. This boy was treated with IVIG and his strength normalized.

An 11-year-old boy presented with three months of weakness to the EDx lab for further evaluation. The physical exam was notable for distal weakness in all limbs, impersistent reflexes, and mild distal sensory loss to all modalities. Ultrasound imaging of the right median nerve was performed as the initial diagnostic test, and revealed multifocal nerve enlargement, increased echogenicity, enlarged fascicles, and increased nerve anisotropy (Figure 1). The median nerve CSA varied between 10 mm^2^ and 20 mm^2^, which is significantly enlarged when compared to normative values in this age group. These ultrasonographic findings increased our suspicion of CIDP. Nerve conduction studies of the right median, ulnar, and tibial nerves subsequently showed significantly prolonged latencies (in the 10 to 24 ms range), slowed velocities (in the 7–18 m/s range), conduction blocks, and a median F wave of 120 ms. He was diagnosed with a severe acquired demyelinating sensorimotor polyneuropathy. The overall presentation was most consistent with CIDP, and he was therefore started on IVIG. He has now completed three courses of IVIG with complete normalization of strength and return to his previous activities. The patient and his mother provided informed consent to include his information in this paper.

## 7. Conclusions

Current literature and experience strongly supports the use of ultrasound in pediatric patients suspected of neuromuscular pathology. More studies investigating nerve and muscle ultrasound in this population are needed, however. Attention to age-related changes of the peripheral nervous system is crucial when interpreting sonographic imaging. Importantly, the advantages associated with neuromuscular ultrasound in the pediatric population should be considered, as this modality can lead to accurate diagnostic evaluations with fewer painful electrodiagnostic tests. Future neuromuscular ultrasound research in the pediatric population should include further defining reference values, exploration of nerve and muscle imaging as biomarkers of disease progression, and the use of new techniques, such as ultra-high resolution and elastography.
